# Prevention Works Best in Pairs: An Observational Study on Connubial Melanoma

**DOI:** 10.3390/diagnostics15151869

**Published:** 2025-07-25

**Authors:** Alessandra Iorio, Maria Concetta Fargnoli, Francesca Sperati, Pasquale Frascione, Paola De Simone

**Affiliations:** 1 Department of Oncological and Preventative Dermatology, San Gallicano Dermatological Institute IRCCS, Via Elio Chianesi, 00144 Rome, Italy; mariaconcetta.fargnoli@ifo.it (M.C.F.); pasquale.frasciopne@ifo.it (P.F.); paola.desimone@ifo.it (P.D.S.); 2 UOSD Clinical Trial Center, Biostatistics and Bioinformatics, San Gallicano Dermatological Institute IRCCS, Via Elio Chianesi, 00144 Rome, Italy; francesca.sperati@ifo.it

**Keywords:** connubial melanoma, couple-based screening, cutaneous melanoma, early detection, environmental risk factors, prevention

## Abstract

**Background:** Connubial melanoma, the occurrence of melanoma in non-consanguineous spouses, is rarely described in the literature. This study aimed to evaluate the prevalence of shared risk factors, preventive behaviors, and the influence of couple dynamics on the early diagnosis of cutaneous melanoma (CM). **Methods:** We conducted a retrospective observational study at the San Gallicano Dermatological Institute IRCCS, Rome, enrolling 52 heterosexual couples diagnosed with CM between 2010 and 2023. Clinical and anamnestic data, including phototype, history of sun exposure, use of tanning devices, and reason for dermatological evaluation, were collected. Dermatological assessments included dermoscopy, total body photography, and histological examination of excised lesions. Statistical analyses were performed using chi-square and Student’s *t*-tests. **Results:** Women reported significantly higher use of artificial ultraviolet sources (51.9% vs. 19.2%, *p* < 0.001) and more frequent histories of sunburn. Phototype II was associated with higher use of tanning devices and a greater prevalence of sunburns. Although the CM stage did not significantly differ between sexes, husbands exhibited a greater Breslow thickness. Melanoma localization differed by sex, with lower limbs more often affected in women and the trunk in men (*p* < 0.001). In 86.5% of cases, wives initiated their husband’s dermatological evaluation, leading to earlier diagnosis. **Conclusions:** Despite shared environmental exposures, men and women differ in preventive behaviors and risk profiles. Women play a crucial role in promoting early detection among couples. Couple-based preventive strategies may be instrumental in improving early melanoma diagnosis and outcomes.

## 1. Introduction

Cutaneous melanoma (CM) pathogenesis is multifactorial: it involves genetic, epigenetic, and environmental factors. Among the susceptibility genetic factors, germline alterations in the *CDKN2A*, *MITF*, *POT1*, and *BAP1* genes are the most recognized cause of CM heritability within populations of different geographic areas; melanoma risk can also be associated with assortative mating on genetic traits, primarily through mate preferences for similar pigmentation traits. This can lead to a non-random distribution of melanoma susceptibility alleles, influencing population-level risk, heritability, and clustering of disease [1].

Among environmental factors, those most notable are linked to individual behavior [2]. Males have approximately twice the risk of developing malignant melanoma compared to females [3].

Irrespective of sex, established risk factors include a family history of melanoma and residence in high-risk geographic regions [4,5], an increased number of nevi—whether typical or atypical [6]—and a light phototype [7]. Among behavioral factors, ultraviolet (UV) radiation remains the most significant environmental risk: chronic and unprotected sun exposure, as well as exposure to artificial sources [6].

Most patients experience only a single episode of CM during their lifetime [2]. Approximately 10% of melanoma patients report a family history of the disease, yet occurrences among non-blood relatives are rarely described in the literature [8]. The term “connubial melanoma” refers to the sporadic development of melanoma in non-consanguineous spouses, unlike familial melanoma, which is characterized by the presence of at least two cases of melanoma among blood relatives and is typically linked to inherited genetic mutations. The anatomical site of melanoma in spouses appears to be random, with few cases reporting melanoma occurring at the same body location in both partners [8]. However, it remains unclear whether connubial melanoma is merely coincidental or influenced by shared environmental risk factors and regular follow-up visits.

We report our experience with 52 couples in which both spouses—wife and husband—were diagnosed with CM.

## 2. Patients and Methods

### 2.1. Study Design and Setting

This study was conducted as a monocentric retrospective observational study at the Department of Oncological and Preventative Dermatology, San Gallicano Dermatological Institute—IRCCS, Rome, Italy. The study was conducted in accordance with the principles of the Declaration of Helsinki and was approved by the Ethics Committee Lazio Area 5 (Ref. N 13730/2024, dated 10 October 2024). All data were handled in compliance with applicable data protection regulations. Identifying information was removed or coded to protect the privacy of individuals. Access to the data was restricted to authorized research personnel, and all analyses were performed on de-identified datasets.

### 2.2. Inclusion Criteria

Eligible participants met the following criteria: (i) age between 18 and 90 years; (ii) literacy; (iii) personal history of primary CM; (iv) spousal partner also diagnosed with CM; (v) patients with sporadic melanoma. All participants were either already under follow-up at the institute or newly referred during the study period.

### 2.3. Clinical and Dermatological Assessment

All patients spontaneously decided to undergo a dermatological visit. They were subjected to a comprehensive clinical evaluation, which included a detailed collection of anamnestic data, such as a history of sunburn during childhood or adolescence, previous diagnoses of CM with corresponding histological subtypes, and lifestyle habits, including the use of tanning beds, sun lamps, and sunscreen. A full dermatological examination was performed, covering the entire skin surface, including the scalp, nails, and mucosal areas. Additionally, each patient was assessed for the number of nevi and signs of prior sun damage.

Dermoscopy and total body photography were used during each clinical evaluation. Any lesions deemed suspicious were surgically excised and submitted for histopathological analysis following the acquisition of informed consent.

### 2.4. Variables Analyzed

We analyzed the following variables for each partner: phototype, histological characteristics and anatomical localization of melanoma, age at diagnosis, number of nevi, history of sunburns, history of exposure to sun lamps, and the reason prompting the dermatological appointment.

### 2.5. Statistical Analysis

Categorical variables were reported as absolute and relative frequencies, while continuous variables were expressed as means with standard deviations (SDs). The Kolmogorov–Smirnov test was used to assess the normality of distribution for all continuous variables. Differences between continuous variables were evaluated using the Student’s *t*-test, and associations between categorical variables were analyzed using the chi-square (χ^2^) test. *p* < 0.05 was considered statistically significant. All statistical analyses were performed using SPSS software, version 29 (IBM Corp., Chicago, IL, USA).

## 3. Results

### 3.1. Study Population

Between 2010 and 2023, we enrolled 52 heterosexual couples in which both spouses had a documented diagnosis of CM. The age of male participants ranged from 39 to 83 years, and that of female participants from 37 to 81 years. The mean age at surgical removal of CM was 53.4 ± 11.5 years for husbands and 50.5 ± 10.9 years for wives.

### 3.2. Comparison of Risk Factors Between Spouses

Table 1 presents a comparison of risk factors between husbands and wives, including history of sunburns, use of tanning beds, presence of multiple nevi, and phototype. A significant difference was observed in the use of artificial UV sources: 27 wives (51.9%) reported using tanning beds or sun lamps, compared to only 10 husbands (19.2%, *p* < 0.001). The other two variables (multiple nevi and phototype) did not show statistically significant differences in the comparison between spouses.

### 3.3. Phototype, Tanning Bed Use, and Sunburn History

Table 2 presents the association between phototype and the use of artificial UV sources in the overall study population. Among patients who reported using tanning beds or sun lamps, 67.6% had phototype II, whereas among those who did not, 43.3% had phototype III (*p* = 0.005). This association remained statistically significant when analyzed separately in the female subgroup (*p* < 0.021). Overall, 70.4% of wives with phototype II reported a history of sunlamp exposure, compared to 60% of husbands with the same phototype. Among individuals with phototype III, 14.8% of women and 20% of men reported exposure to artificial UV sources.

Additionally, Table 2 shows a significant association between phototype and history of sunburns in the overall population (*p* < 0.001). Specifically, 62.5% of individuals with a history of sunburns had phototype II, while 70.8% of those without sunburns had phototype III. When analyzed separately, 54.8% of husbands and 71.0% of wives with a history of sunburns had phototype II.

### 3.4. Stage and Anatomical Distribution of Melanoma

As shown in Table 3, there were no differences between men and women in terms of CM stage (*p* = 0.24). However, Breslow thickness tended to be higher in husbands, with 9.6% presenting with pT1b stage melanoma compared to 5.8% of wives.

The thin Breslow thickness melanoma observed in both groups is associated with a 100% survival rate in this cohort.

A significant difference was observed in the anatomical distribution of melanoma onset between spouses: among wives, the lower limbs were the most frequently affected site (67.4%), whereas in husbands, the trunk was predominantly involved (63.4%; *p* < 0.001). In contrast, only 21.2% of husbands had melanomas on the lower limbs, and 19.2% of wives had involvement of the trunk.

### 3.5. Initiative in Seeking Dermatological Evaluation

Additionally, anamnestic data revealed that in 45 out of the 52 couples (86.5%), it was the wife who prompted her husband to undergo dermatological evaluation. In these cases, the women had previously undergone surgical removal of a melanoma, either recently or in the more distant past. During the husband’s visit, the dermatologist identified a suspicious lesion, which was subsequently confirmed as melanoma upon histological examination.

In the remaining seven couples (13.4%), both spouses attended the dermatological visit together—again, at the wife’s initiative—and were diagnosed synchronously with melanoma.

Only in one couple did the husband, already diagnosed with melanoma, encourage his wife to undergo evaluation, which led to the surgical removal and histological confirmation of melanoma.

## 4. Discussion

According to our data, the main risk factors among married couples are linked to lifestyle habits established during childhood and reinforced by shared behaviors throughout their life together. Phototype II was more frequent among users of artificial UV sources and was significantly associated with a history of sunburn. Melanoma site distribution varied by sex, with the lower limbs more commonly affected in women and the trunk in men, although husbands tended to present with thicker melanomas. A key finding in our cohort is that, in most cases, it was the wives who prompted their husbands to undergo dermatological evaluation, leading to diagnosis.

Anamnestic data indicate that spouses are generally exposed to the same environmental risk factors. However, men and women tend to differ in their individual approaches to prevention; the women have an evident advantage: this seems to depend on both biological sex traits and behavioural differences regarding primary prevention (sun exposure, UV-protection) and secondary prevention as skin screening [9].

Within the couple dynamics, the wife can make a difference.

Although connubial melanoma is considered a rare phenomenon in the literature, our findings suggest that it may be more common than previously recognized.

Connubial melanoma represents a coincidental occurrence among non-blood relatives, likely influenced by shared exposure to environmental risk factors during both childhood/adolescence and married life. Many of the patients in our study reported similar sun-related behaviors during their youth, such as spending prolonged periods outdoors during summer with intense, unprotected sun exposure and a history of sunburns—often due to limited awareness of the risks associated with UV radiation. The use of sunscreen among almost all male partners was found to be sporadic and inconsistent. Women are more likely to use sunscreens mainly during sun exposure.

In addition to natural sun exposure, the use of tanning beds was frequently reported among participants during adolescence and young adulthood (ages 15–30 years). There are several types and denominations of tanning devices (sunbeds, tanning beds/booths/canopies, and solariums): the term “sunbeds” is commonly used to collectively define them all [9]. In 2020, the International Agency for Research on Cancer (IARC) classified artificial tanning devices as carcinogenic and included them in the highest-risk category [10], as tanning beds can emit UVA radiation at doses up to 12 times greater than natural sunlight [6].

This behavioral pattern often persists into adulthood and is shared by many couples, particularly those who have cohabited for over 20 years. Nevertheless, our anamnestic data show distinct gender-related differences in UV exposure. Women tend to engage in more controlled sun exposure and are more likely to use photoprotective cosmetic products. However, they are also more inclined than men to use sun lamps and tanning beds, potentially increasing their risk of melanoma. In contrast, husbands generally report lower usage of sunscreen and artificial tanning devices, indicating poorer primary prevention efforts and potentially contributing to the development of melanomas with greater Breslow thickness.

Furthermore, our data suggest that individuals with phototype II are more likely to use artificial UV sources compared to those with phototype III. As a result, they report a higher frequency of sunburns and often exhibit clinical signs consistent with previous UV-induced damage, which accelerates premature aging and heightens the risk of skin cancer [11].

Self-examination plays a critical role in the early detection of CM, yet notable differences exist between men and women in preventive behavior. Women demonstrate a greater inclination toward prevention; they are more likely to undergo regular dermatological evaluations and participate in mass screening campaigns conducted by trained dermatologists. They also tend to engage more consistently in self-examinations—one of the simplest and most accessible methods for early melanoma detection.

Consistent with findings from the literature [9,12,13], our data show that the most common anatomical sites of melanoma onset correspond to areas exposed to intermittent and intense sun exposure. Among women, the lower limbs are most frequently affected, whereas in men, the trunk is the predominant site.

Regarding survival outcomes, the wives and their husbands in our melanoma patient population currently exhibit comparable survival rates. This can be attributed to the generally thin Breslow thickness observed in both groups, likely a result of our proactive skin cancer prevention and screening efforts, which have enabled early diagnosis and contributed to a 100% survival rate in this cohort.

In our cohort of couples, women frequently played a decisive role in prompting their husbands to attend dermatological follow-up visits [14]. Many husbands began their consultations with remarks such as, “My wife booked my visit,” or “Thanks to my wife, I removed the melanoma.” This underscores the heightened attentiveness women often demonstrate toward their partner’s health. They not only encourage medical checkups but also accompany their husbands to visits, having often noticed “alarming” or “changing” lesions on their skin. As a result, women may receive diagnoses at earlier stages, whereas men—without this encouragement—might face later detection and treatment. Literature data have shown that unmarried patients with cancer have a higher risk of being diagnosed with cancer at an advanced stage of the disease than married patients and that marriage may play a greater protective role in the cancer-specific survival of men than of women [15,16].

Given that shared environmental risk factors can contribute to melanoma development in both partners, regular skin self-examination and periodic dermatological follow-ups remain the most effective strategies for early diagnosis. Furthermore, sunscreen use is recommended for sun protection in addition to clothing and shade [11,17].

Both partners in couples, after a melanoma diagnosis, report that they have stopped using tanning lamps, reduced prolonged unprotected sun exposure, and increased the frequency of sunscreen use, along with a greater inclination toward prevention.

The findings of this study support future research aimed at understanding how interpersonal dynamics influence melanoma prevention and early detection. Prospective, multicenter studies could further investigate the impact of partner involvement in promoting timely dermatological assessment and early diagnosis. Additionally, as the present study focuses exclusively on heterosexual couples, it would be interesting to investigate other relationship dynamics and more diverse populations to determine whether the trends observed in this study are consistent across varying cultural, social, and relational settings.

### Study Limitations

Given the retrospective observational design of the study, several limitations should be acknowledged. The findings may be subject to selection bias, as the population includes only patients who spontaneously presented to our center for dermatological evaluation. Additionally, recall bias may affect the accuracy of self-reported information regarding sun exposure, tanning habits, and history of sunburns. These factors should be considered when interpreting the results.

Furthermore, the absence of a control group limits the ability to assess relative risk or establish causality and may reduce the generalizability of the findings. Due to the relatively small sample size, it was not feasible to perform additional subgroup analyses, such as those based on duration of marriage or combined gender–phototype categories, without compromising statistical robustness. Finally, given that the primary aim of the study was to compare husbands and wives diagnosed with melanoma in order to identify potential differences in lifestyle and risk profiles, univariate analysis was considered the most appropriate and informative approach, and no multivariable analyses were performed.

## 5. Conclusions

In the context of melanoma prevention within couples, women may have a particularly active role in promoting health. Due to their traditional social role as the cornerstone of family relationships, women often encourage healthy behaviors and lifestyle choices, both for themselves and their loved ones. Their receptiveness, proactivity, and empathy can make them particularly attuned to preventive healthcare, helping to engage the entire family in maintaining well-being.

As the majority of family caregiving responsibilities fall to women, it is essential to recognize and analyze the profound value of their role—not only in society at large but also possibly within the framework of disease prevention and health promotion.

## Figures and Tables

**Table 1:** Comparison of melanoma-related risk factors between husbands and wives in 52 connubial melanoma couples.

Variable	Husbands (*n* = 52)		Wives (*n* = 52)		χ^2^ Test (*p*-Value)
Exposure to artificial UV sources	*n* (%)	95% CI	*n* (%)	95% CI	
No	42 (80.8%)	0.69–0.90	25 (48.1%)	0.37–0.63	<0.001
Yes	10 (19.2%)	0.10–0.31	27 (51.9%)	0.37–0.63	
History of sunburns					
No	10 (19.2%)	0.10–0.31	14 (26.9%)	0.16–0.39	0.352
Yes	42 (80.8%)	0.69–0.90	38 (73.1%)	0.61–0.84	
Presence of multiple nevi					
No	22 (42.3%)	0.29–0.54	25 (48.1%)	0.37–0.63	0.554
Yes	30 (57.7%)	0.46–0.71	27 (51.9%)	0.37–0.63	
Phototype					0.718
I	5 (9.6%)	0.04–0.20	5 (9.6%)	0.04–0.20	
II	23 (44.3%)	0.30–0.56	27 (51.9%)	0.41–0.66	
III	18 (34.6%)	0.25–0.50	17 (32.7%)	0.21–0.45	
IV	6 (11.5%)	0.05–0.22	3 (5.8%)	0.02–0.15	

*p* < 0.05 = statistically significant.

**Table 2:** Association between phototype and exposure to artificial UV sources or history of sunburns in the overall population and by sex (*n* = 104).

Phototype	Overall (*n* = 104)					Husbands (*n* = 52)				Wives (*n* = 52)			
	*n* (%)		95% CI	*n* (%)	95% CI	*n* (%)	95% CI	*n* (%)	95% CI	*n* (%)	95% CI	*n* (%)	95% CI
Phototype by use of artificial UV sources	No (*n* = 67)			Yes (*n* = 37)		No (*n* = 42)		Yes (*n* = 10)		No (*n* = 25)		Yes (*n* = 27)	
I		5 (7.5%)	0.03–0.15	5 (13.5%)	0.06–0.28	3 (7.1%)	0.02–0.18	2 (20.0%)	0.06–0.51	2 (8.0%)	0.02–0.23	3 (11.1%)	0.04–0.28
II		25 (37.3%)	0.28–0.50	25 (67.6%)	0.51–0.80	17 (40.5%)	0.26–0.53	6 (60.0%)	0.31–0.83	8 (32.0%)	0.21–0.56	19 (70.4%)	0.51–0.84
III		29 (43.3%)	0.33–0.55	6 (16.2%)	0.08–0.31	16 (38.1%)	0.28–0.55	2 (20.0%)	0.06–0.51	13 (52.0%)	0.31–0.66	4 (14.8%)	0.06–0.32
IV		8 (11.9%)	0.06–0.21	1 (2.7%)	0.00–0.14	6 (14.3%)	0.06–0.27	0 (0.0%)	0.00–0.28	2 (8.0%)	0.02–0.23	1 (3.7%)	0.01–0.18
*p*-value				0.005				0.233				0.021	
Phototype by history of sunburns	No (*n* = 24)			Yes (*n* = 80)		No (*n* = 10)		Yes (*n* = 42)		No (*n* = 14)		Yes (*n* = 38)	
I	0 (0.0%)		0.00–0.14	10 (12.5%)	0.07–0.20	0 (0.0%)	0.00–0.28	5 (11.9%)	0.05–0.24	0 (0.0%)	0.00–0.21	5 (13.2%)	0.05–0.26
II	0 (0.0%)		0.0–0.14	50 (62.5%)	0.51–0.71	0 (0.0%)	0.00–0.28	23 (54.8%)	0.38–0.66	0 (0.0%)	0.00–0.21	27 (71.0%)	0.57–0.84
III	17 (70.8%)		0.51–0.85	18 (22.5%)	0.16–0.34	5 (50.0%)	0.24–0.76	13 (30.9%)	0.22–0.49	12 (85.7%)	0.60–0.96	5 (13.2%)	0.05–0.26
IV	7 (29.2%)		0.15–0.49	2 (2.5%)	0.01–0.08	5 (50.0%)	0.24–0.76	1 (2.4%)	0.00–0.12	2 (14.3%)	0.04–0.40	1 (2.6%)	0.00–0.13
*p*-value				<0.001				<0.001				<0.001	

*p* < 0.05 = statistically significant, chi-square test.

**Table 3:** Tumor stage and anatomical site of melanoma onset in husbands and wives (*n* = 52 couples).

Variable	Husbands (*n* = 52)		Wives (*n* = 52)	
Tumor stage	*n* (%)	95% CI	*n* (%)	95% CI
pTis	23 (44.3%)	0.31–0.57	32 (61.5%)	0.47–0.72
pT1a	21 (40.4%)	0.29–0.55	17 (32.7%)	0.23–0.47
pT1b	5 (9.6%)	0.04–0.20	3 (5.8%)	0.02–0.15
pT2a	2 (3.8%)	0.01–0.13	0 (0.0%)	0.00–0.07
pT3/4	1 (1.9%)	0.00–0.10	0 (0.0%)	0.00–0.07
Total (*p* = 0.249)	52 (100%)		52 (100%)	
Anatomical site				
Abdomen/back	33 (63.4%)	0.51–0.76	10 (19.2%)	0.12–0.33
Upper/lower limbs	11 (21.2%)	0.12–0.33	35 (67.4%)	0.52–0.77
Pectoral region	3 (5.8%)	0.02–0.15	1 (1.9%)	0.00–0.10
Head/neck	5 (9.6%)	0.04–0.20	6 (11.5%)	0.05–0.22
Total (*p* < 0.001)	52 (100%)		52 (100%)	

*p* < 0.05 = statistically significant, chi-square test.

## Data Availability

The data that support the findings of this study are available from the corresponding author upon reasonable request.
